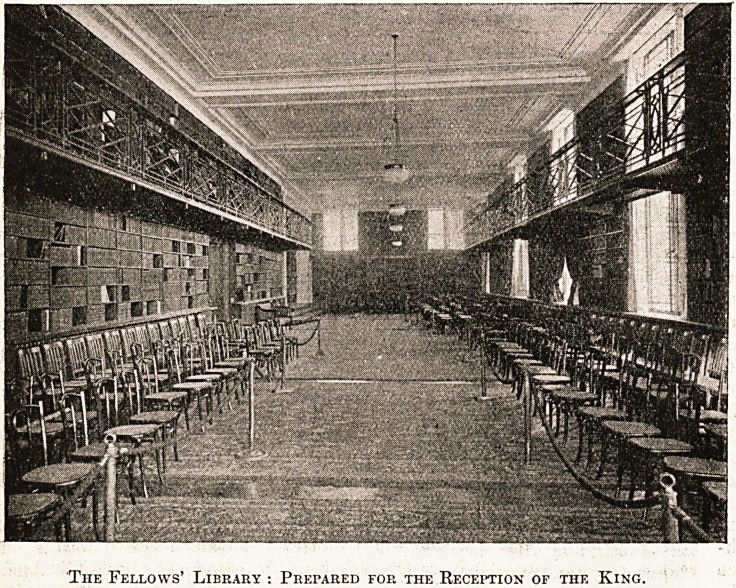# No. 1 Wimpole Street: The New Premises of the Royal Society of Medicine

**Published:** 1912-05-25

**Authors:** 


### No. 1 WIMPOLE STREET.

### The New Premises of the Royal Society of Medicine.

ON Tuesday last, as we note elsewhere, the King and Queen opened the new house of the Royal Society of Medicine, which will be known in the Post Office Directory as 1 Wimpole Street, though the entrance door and the front of the building are in Henrietta Street round the corner. Anyone who approaches from Cavendish Square, the new building which Messrs. Belcher and Joass have designed, cannot fail to be struck by the contrast between the massive front of Portland stone, treated in free Renaissance style, and the yellow bricks of the east side of the building. There, if nowhere else, Messrs. Belcher and Joass have had evidently to study economy.

The front door opens into a large hall, which is simple and dignified, on the right of which are the Robert Barnes lecture hall, with seating accommodation for three or four hundred people, and several cloak rooms. Future speakers from the rostrum, and future audiences, will both benefit by the raised floor, which here and in the principal lecture rooms, of which there are two, are noteworthy improvements. The space occupied by the Robert Barnes lecture hall, on the right as you enter, is filled on the left by a cloak room, a smaller lecture room, and at the far end by an open angle of the hall, which leads to a group of rooms for the examination of cases. This angle not only robs the hall of formality and stiffness, but contains a beautiful eighteenth-century marble mantlepiece (of which more will be written later), surmounted by a presentation clock, the gift of the President, Sir Henry Morris, Bart., to commemorate the opening day. Opposite to the front door is the lift and staircase.

On the first floor is the fine Fellows' Library, an immense and stately room which runs the whole length of the front, and rather more, for it gains in beauty and size from a short arm running back from the street, and making the library the shape of an L. To correspond with this annexe, but on the western or Wimpole Street side of the building, is a large mezzanine room. We are tempted further to describe the library, but presumably all the Fellows will shortly make their own acquaintance with it, and that part of the general hospital public, with an instinct for the proper housing of books, may well envy them their acquisition. On either side of the lift, and divided from the library by the midway corridor, are the pamphlet room, the room for the Librarian, Mr. Hewitt, and the Fellows' lavatory. Parquet floors, new oak doors and woodwork, electric light, and Turkey carpets combine to produce the atmosphere in which the Society's activities will be carried on.

If we pursue our researches to the second floor we find on the extreme left as we leave the lift the Council Chamber, with its large horse-shoe table, red leather chairs, and the chair of honour, surmounted by the Royal Arms, for the use of the President. The Council Chamber, like all the other rooms, is luxurious, though at present engagingly simple, and is specially noteworthy for containing the only piece of pure decoration and the first of the two examples of fine art which the building contains—the Bacon plaque over the fireplace. Its history, if not its quality, is contained in the printed inscription beneath it, which reads as follows:—

THE NEW HOME OF THE ROYAL SOCIETY OF MEDICINE.

"This medallion is the original model by John Bacon, R.A., representing Æneas escaping from burning Troy carrying his father, the blind Anchises. His son, Ascanius, is clinging to his skirts, and Creusa, the wife of Æneas, follows behind.

"For this medallion Bacon was awarded the Gold Medal of the Royal Academy in the year of its foundation, 1768.

"It was purchased by Sir William Chambers, one of the founders and first President of the Academy, and was placed by him over the dining-room mantlepiece of the house he was at that time building for himself at 53 Berners Street. The house afterwards became the home of the Royal Medical and Chirurgical Society. The medallion was removed along with the mantlepiece in 1889 by

Mr. MacAlister and placed in the Society's new house at 20 Hanover Square. In 1912 they were again removed to the Society's present house and the medallion fixed in the Council Room and the mantlepiece in the entrance hall."

Here, too, is the reference to the hall mantlepiece promised above. Opening out of the Council Chamber, and nearer the centre of the second floor, is a vestibule, parallel to which, and still nearer to the west or Wimpole Street side of the building, are three more. Here, too, exactly opposite the lift, is the Henry Louis Florence room, the door of which has been, perhaps temporarily, removed. Beyond this, and with windows overlooking Wimpole Street, is a large committee room, unlike the lecture rooms, with a flat floor. Opposite to the Council Chamber, and behind it, are open flats, which perform a double function. The lesser is to provide space for the erection of a refreshment tent, a convenience easily realisable on such gala days as the Royal opening this week by all who have experience of arranging such affairs. The greater or primordial function of the flats is to light the third floor. Had they been roofed in permanently it is difficult at first glance to see how this could have been done. The fact that they do provide an admirable light allows the corresponding space to them on the other and western side of the lift to be devoted to the President's room. Here, again, rather more elaborate furniture apart, the only decoration is the President's portrait, Sir Henry Morris by an unknown artist, one—we mean—whose name was not discernible. On this floor also, overlooking Wimpole Street, are the Fellows' tea room and smoking room, with the customary telephone and sofas, and the added convenience of a kitchen lift. Tea and light refreshments are expected to be the only meals which the Fellows will require.

The Fellows' Library : Prepared for the Reception of the King.

The third floor is divided between the laboratory, the lady Fellows' room, and the secretarial offices. Over the Council Chamber is the Marcus Beck laboratory, at present containing merely a power wheel, washbasins, and a bookcase. If a question may be permitted it would be to ask whether the colour of the linoleum on the laboratory floor will prove as little dazzling to the eyes of future workers as the colours of the carpets in less workaday rooms. A good view of the surface bricks at the back can be obtained from the laboratory windows. Opposite to the lift is a congeries of rooms with a common door dividing them from the corridor. It comprises the quarters of the Secretary, Mr. MacAlister, who can lock his own door at fifteen feet away by pressing a knob in the wall. The unit includes the cashier's room, and by a second door Mr. MacAlister has access to a lecture or meeting room at the Wimpole Street corner. Miss Williamson's room is also on this floor. At the extreme back, and above the Fellows' smoking room, is a second unit—the lady Fellows' quarters, chief of which is a single and pleasantly sized room, with a beautiful green Wilton. Contiguous to the lady Fellows' unit, but nearer to the lift, is Dr. Nachbar's room. This exhausts the third floor and the main building. A word as to the basement should be added. It is very large, and besides containing the kitchen and porter's room, which opens into the back area, whence the fire escape staircase ascends, the whole extent of the rear of the building is given up principally to an additional library store.

Of the extensive cloak rooms and lavatories in the basement no further need be said, but we cannot refrain from praising the bookcases. They are of iron, and ugly perhaps to look at, but the simple ingenuity by which each shelf can be raised to fit the size of any book is a joy to anyone who has ever had to lament that the old terms folio, quarto, and duodecimo no longer represent standard sizes, while numberless shapes and intermediate patterns, from thumb prayer-books to publications the shape of a cheque on its end, have made the designing of bookshelves not merely a lost art, but an art impossible to recover. This American patent is the nearest attempt we have seen at a simple solution.

## Figures and Tables

**Figure f1:**
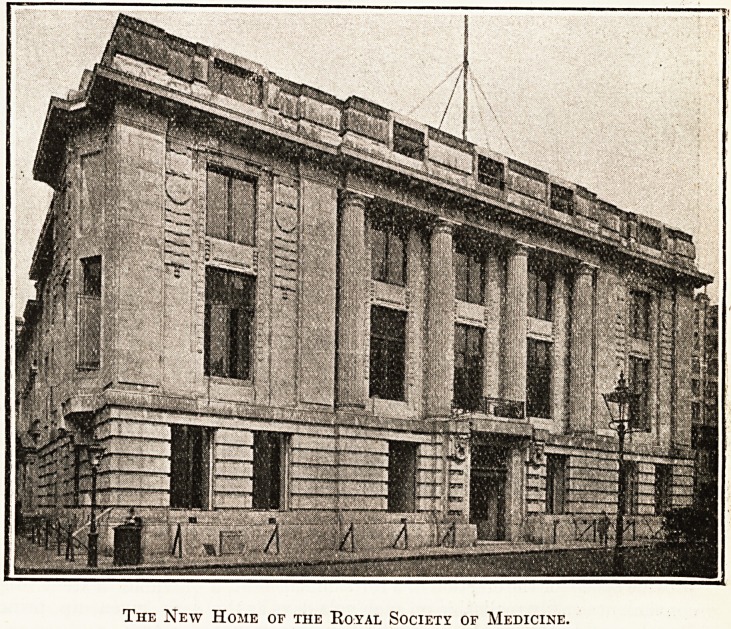


**Figure f2:**